# The Construction of the Self-Induced Sal System and Its Application in Salicylic Acid Production

**DOI:** 10.3390/molecules28237825

**Published:** 2023-11-28

**Authors:** Xin Jin, Yaping Gao, Xuanmu Chen, Sumeng Wang, Qingsheng Qi, Quanfeng Liang

**Affiliations:** State Key Laboratory of Microbial Technology, Shandong University, Qingdao 266237, China; sdu_jinxin@outlook.com (X.J.); 15849607404@163.com (Y.G.); woshicxm1993@163.com (X.C.); smwang001@126.com (S.W.); qiqingsheng@sdu.edu.cn (Q.Q.)

**Keywords:** quorum sensing system, synthetic biology, salicylic acid, artificial trans-encoded sRNAs, metabolic engineering

## Abstract

The design and construction of more complex and delicate genetic control circuits suffer from poor orthogonality in quorum sensing (QS) systems. The Sal system, which relies on salicylic acid as a signaling molecule, is an artificially engineered regulatory system with a structure that differs significantly from that of natural QS signaling molecules. Salicylic acid is an important drug precursor, mainly used in the production of drugs such as aspirin and anti-HIV drugs. However, there have been no reports on the construction of a self-induced Sal system in single cells. In this study, a high-copy plasmid backbone was used to construct the regulatory proteins and a self-induced promoter of salicylic acid in *E. coli* by adjusting the precise regulation of key gene expression; the sensitivity and induction range of this system were improved. Subsequently, the exogenous gene *pchBA* was introduced in *E. coli* to extend the shikimate pathway and synthesize salicylic acid, resulting in the construction of the first complete self-induced Sal system. Finally, the self-induced Sal System was combined with artificial trans-encoded sRNAs (atsRNAs) to repress the growth-essential gene *ppc* and accumulate the precursor substance PEP, thereby increasing the titer of salicylic acid by 151%. This construction of a self-induced artificial system introduces a new tool for selecting communication tools and induction systems in synthetic biology and metabolic engineering, but also demonstrates a self-inducible pathway design strategy for salicylic acid biosynthesis.

## 1. Introduction

QS is a bacterial cellular signaling mechanism that is dependent on population density [1,2]. Within the field of synthetic biology, the QS system is extensively studied as a means of communication and self-regulation. Through advancements in synthetic biology and metabolic engineering, QS systems have been utilized in gene circuit design, synthetic system construction, and product production [3,4,5,6,7]. For example, our group has used QS to create logic gates and bifunctional dynamic switches (QS switches) for the synthesis of poly (β-hydroxybutyric acid) (PHB) [8,9] and 5-aminolevulinic acid (ALA) [8], addressing important metabolic engineering challenges. Xu et al. have also employed the QS system as a communication module in the construction of a microbial ecosystem [10].

QS systems are commonly used in synthetic biology as tools for intercellular communication. Among these, the LuxI/R QS system stands out due to its simplicity and natural diversity, utilizing AHL as a signaling molecule [11,12,13,14]. However, the receptor proteins in LuxI/R QS systems are structurally similar and the chemical structures of the signaling molecules are also similar, leading to significant crosstalk between different QS systems [15,16]. Moreover, some AHL synthetases produce non-specific signaling molecules, further challenging the orthogonality of the systems. This crosstalk between QS systems can result in unintended outcomes when multiple systems coexist in the same environment [17,18]. The development of synthetic biology has led to the construction of complex QS regulatory networks and circuits that use multiple receptor proteins to detect different signals and interact. Proper intercellular communication requires well-insulated signaling channels [19], as crosstalk between components can disrupt system functionality [17,18]. Therefore, the design and construction of crosstalk-free QS systems are crucial for the development of more complex and precise genetic control circuits. Although some progress has been made in this field, such as our group’s construction of an orthogonal QS system in *E. coli* that satisfies both promoter and signaling orthogonality [20], existing orthogonal QS systems are still inadequate and further advancements are needed to expand the applications of QS systems.

Salicylic acid is a highly valuable compound [21] commonly synthesized through chemical methods which result in environmental pollution [22]. However, recent research has explored the possibility of microorganism-based synthesis of salicylic acid [23,24,25]. Various natural transcription factors, such as CmeR and NahR, respond to salicylic acid in organisms. Due to its unique molecular structure, salicylic acid can be designed as a novel inducer for cellular communication and regulation, showing potential as a communication system for co-culture applications [19]. For example, salicylic acid can be synthesized in *Saccharomyces cerevisiae* and sensed in *E. coli* to facilitate information exchange among microorganisms [19]. Other studies have focused on the response of NahR to salicylic acid, an intermediate in the catabolic pathway of the environmental pollutant naphthalene. Researchers have designed NahR and P_sal_, a promoter that responds to salicylic acid, to sense the amount of naphthalene in the environment [26]. Additionally, biosensors responsive to salicylic acid and various aromatic and indole inducers have been constructed in model organisms using transcription factor CmeR from *Campylobacter jejuni* and belonging to the TetR family of repressors [27]. However, current salicylic acid-based induction systems typically rely on exogenous additions or heterologous supplies of inducers, limiting their practical applications. Therefore, it is crucial to develop a complete self-induced salicylic acid regulatory system within a single cell. Moreover, the Sal system, with its simplified synthase composition and unique signaling molecular structure that differs from natural Las and Lux QS systems, has the potential to construct a completely orthogonal QS system.

**Figure 1:**
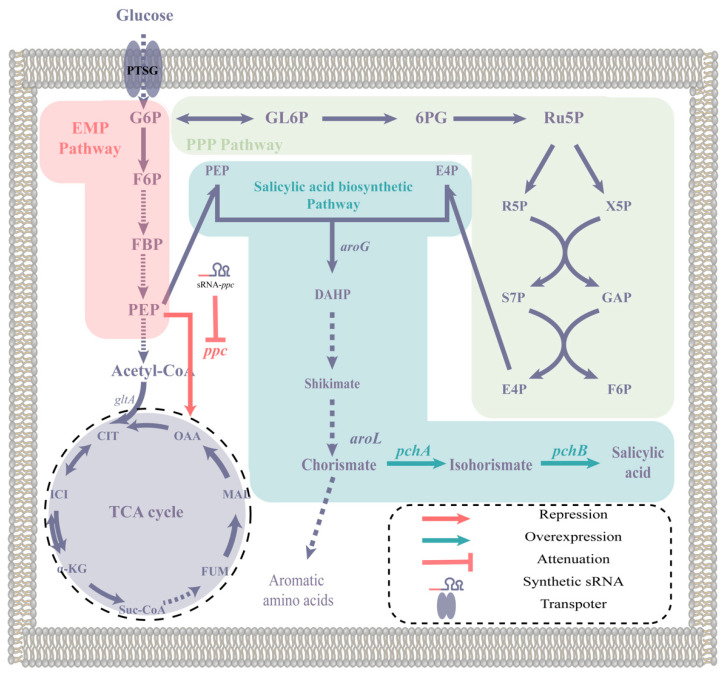
The biosynthetic pathway of salicylic acid and the modification strategy of this study. The abbreviations used in this paragraph are as follows: G6P for glucose 6-phosphate, GL6P for 6-phosphogluconolactone, 6PG for 6-phosphogluconate, Ru5P for ribulose 5-phosphate, R5P for ribose 5-phosphate, X5P for xylulose 5-phosphate, S7P for sedoheptulose 7-phosphate, GAP for glyceraldehyde 3-phosphate, E4P for erythrose 4-phosphate, F6P for fructose 6-phosphate, FBP for fructose bisphosphate, PEP for phosphoenolpyruvate, OAA for oxaloacetate, CIT for citrate, ICI for isocitrate, α-KG for α-ketoglutarate, Suc-CoA for succinyl-CoA, FUM for fumarate, MAL for malate, and DHAP for 3-deoxy-D-arabinoheptulosonate-7-phosphate. The genes mentioned in this paragraph are as follows: *aroG* encodes 3-deoxy-D-arabinoheptulosonate-7-phosphate (DHAP) synthase, *aroL* encodes shikimate kinase, *ppc* encodes phosphoenolpyruvate carboxylase, *pchA* encodes isochorismate synthase, and *pchB* encodes isochorismate pyruvate lyase.

In this study, the Sal QS system was constructed in *E. coli* for the first time by integrating genes responsible for salicylic acid synthesis and regulatory proteins along with a self-induced promoter. By making precise adjustments to the regulation of key genes, the sensitivity and range of induction of this system were improved. Finally, the self-induced Sal system was utilized for the production of salicylic acid by combining it with artificial trans-encoded sRNAs (atsRNAs) (Figure 1).

## 2. Results and Discussion

### 2.1. Design and Construction of the Self-Induced Sal System

**Figure 2:**
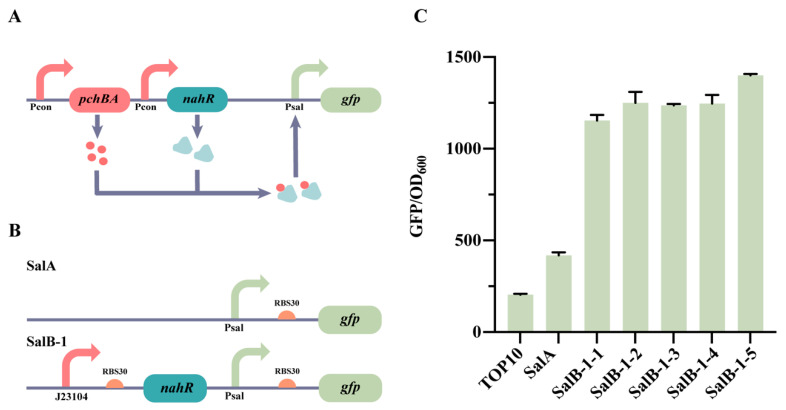
Illustration of the construction of the Sal QS system. (**A**) The working principle of the Sal QS system. (**B**) The diagram displays the pattern of the Sal QS system. (**C**) SalA and SalB-1 are characterized in this figure. SalB-1-1, SalB-1-2, SalB-1-3, SalB-1-4, and SalB-1-5 are evaluated for fluorescence by adding varying concentrations of salicylic acid (0, 10^−6^, 10^−5^, 10^−4^, and 10^−3^ mM), while the remaining strains are induced with 0 mM salicylic acid. The data presented represent the mean with standard deviation (*n* = 3 independent experiments).

Figure 2A shows the design of the Sal system which is used to develop an orthogonal artificial QS system. This system utilizes the *pchBA* genes from *Pseudomonas aeruginosa* as a synthase gene, the *nahR* gene from *Pseudomonas malodorata* as a transcription factor coding gene, and the P_sal_ promoter. The products of *pchBA* genes synthesize salicylic acid using branched-chain amino acids as a precursor, and the combination of salicylic acid and NahR proteins forms a complex that activates GFP expression. In a study by Du et al., a Sal sensing system in *E. coli* is constructed, but it requires an additional signaling molecule. The authors discover that NahR has low sensitivity to salicylic acid and conduct random mutations on NahR to find a mutant with higher sensitivity called NahR^Q^[168]^R^ [19].

We started by choosing the NahR^Q^[168]^R^ as the transcription factor and constructing the Sal system with an added salicylic acid on a high-copy plasmid (Figure 2B). The strain, named SalB-1, was created by expressing NahR^Q^[168]^R^ under the control of a P_sal_ promoter to regulate GFP expression (Figure 2B). In the control strain, SalA, GFP expression was solely controlled by a P_sal_ promoter (Figure 2B). The SalB-1 strain was then evaluated for fluorescence by adding varying concentrations of salicylic acid (0, 10^−6^, 10^−5^, 10^−4^, and 10^−3^ mM). The control strains used were *E. coli* TOP10 and SalA, and the results are depicted in Figure 2C. The fluorescence intensity of SalA was found to be twice as high as that of *E. coli* TOP10, suggesting some leakage from the P_sal_ promoter. When different concentrations of the salicylic acid were added to SalB, no significant changes in fluorescence intensity were observed (Figure 2C). This indicates the possibility of a mismatch in the expression of NahR^Q^[168]^R^ or GFP in the Sal system. To address this issue, modifications were made to the Sal system, primarily focusing on adjusting the expression level of NahR.

### 2.2. Optimization of the Self-Induced Sal System

**Figure 3:**
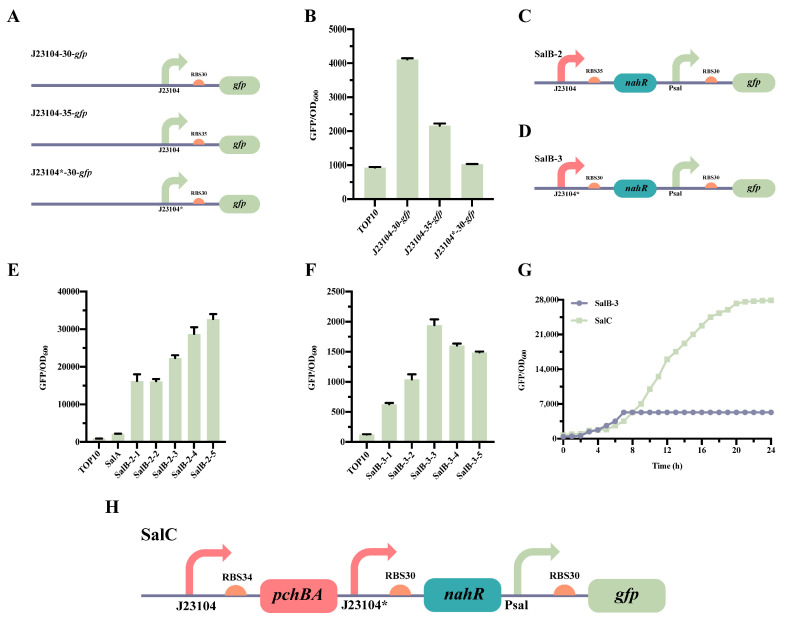
Optimization of the Sal QS system. (**A**) Pattern diagram of different RBS and promoter substitutions in the Sal QS system. (**B**) Characterization of various RBSs and promoter strengths. (**C**) Plasmid design for the optimized SalB-2 Sal QS systems. (**D**) Plasmid design for the optimized SalB-3 Sal QS systems. (**E**) Characterizations of SalB-2. SalB-2-1, SalB-2-2, SalB-2-3, SalB-2-4, and SalB-2-5 were evaluated for fluorescence by adding varying concentrations of salicylic acid (0, 10^−6^, 10^−5^, 10^−4^, and 10^−3^ mM), and the remaining strains were induced with a 0 mM salicylic acid. (**F**) Characterizations of SalB-3. SalB-3-1, SalB-3-2, SalB-3-3, SalB-3-4, and SalB-3-5 were evaluated for fluorescence by adding varying concentrations of salicylic acid (0, 10^−6^, 10^−5^, 10^−4^, and 10^−3^ mM), and the remaining strains were induced with a 0 mM salicylic acid. (**G**) Characterization of the Sal QS system. (**H**) Design of a plasmid for the SalC system, which incorporates the *pchBA* genes to create a self-induced system. The data presented in this figure represent the mean ± SD of three independent experiments.

SalB-2 was developed to modify the sensitivity of the Sal system towards salicylic acid by replacing the RBS of SalB-1 with BBa_B0035 (http://parts.igem.org/, accessed on 9 April 2021) [28] and adjusting the expression level of NahR. During the construction and characterization of SalB-2, we discovered that one strain, SalB-3, underwent mutation. Sequencing confirmed that the promoter J23104 of SalB-3 mutated to J23104* (Table S4). Different promoters and RBS sequence combinations (23104-RBS30, J23104-RBS35 and J23104*-RBS30), respectively, control the expression of NahR to form strains SalB-1, SalB-2 and SalB-3 (Figure 3A). The results of the characterization demonstrate that SalB-1, SalB-2, and SalB-3 control the level of NahR expression in the following order, from high to low: J23104-RBS30 > J23104-RBS35 > J23104*-RBS30 (Figure 3B).

The characterization of SalB-2 (Figure 3C) and SalB-3 (Figure 3D) revealed that the Sal system in SalB-2, which had RBS35 replaced, showed relatively low sensitivity to salicylic acid. It had a response range of only 1.1-fold with the addition of 0 mM to 10^−3^ mM salicylic acid (Figure 3E). On the other hand, SalB-3, which had a mutated promoter J23104*, was sensitive to salicylic acid induction, with a range of threefold when a 0 mM to 10^−3^ mM salicylic acid was added (Figure 3F). Additionally, SalB-2 had a leakage value of 16,000, much higher than the leakage value of SalB-3, which was 630. Therefore, SalB-3, which performed better, was chosen for the subsequent step in constructing the Sal system.

The construction of strain SalC involved adding the synthase gene *pchBA* to SalB-3, resulting in the complete self-induced system (Figure 3H). Strains SalB-3 and SalC were then characterized in 24-well plates. After culturing at 37 °C under vigorous shaking, the cell density at OD_600_ and green fluorescence were detected using a Multi-Detection Microplate Reader. The results revealed that GFP was expressed in the SalC strain, unlike in the SalB-3 strain (Figure 3G). This means that SalC is capable of synthesizing salicylic acid through induced reporter gene expression. Consequently, the complete self-induced Sal system was successfully established in *E. coli* TOP 10, thus expanding the tool library of synthetic biology QS systems.

During practical applications, the QS system encounters the issue of poor orthogonality [29]. In order to address this problem, synthetic biologists replicated the structure of the QS system and developed several effective artificial QS systems. These systems were created by designing the biosynthetic pathway of signaling molecules, making rational modifications to the inducible promoter, and strategically evolving transcription factors, among other measures [15,30,31,32].

The majority of the signaling molecules used in these systems are secondary metabolites, which have a different structure compared to the signaling molecules in the traditional QS system. Moreover, these secondary metabolites exhibit improved orthogonality, providing more options for applications based on QS.

### 2.3. Construction of the atsRNAs That Combine with the Self-Induced Sal System

**Figure 4:**
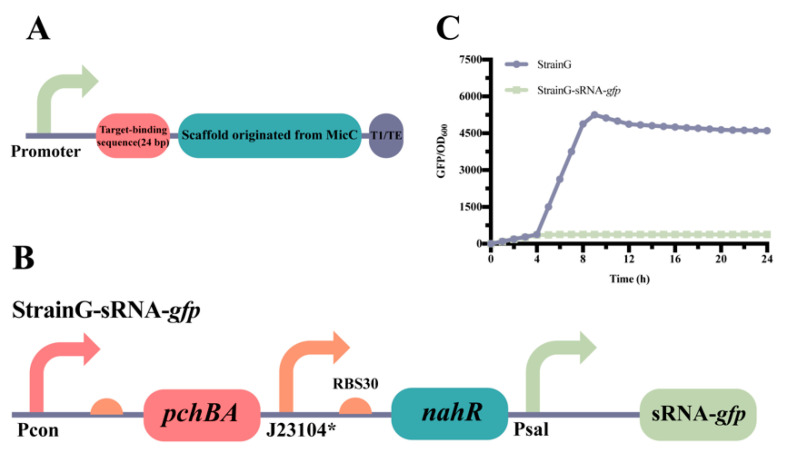
Construction and characterization of atsRNA. (**A**) The genetic structure of atsRNA, featuring T1/TE as the transcriptional terminator (MITRegistry BBa_B0025). (**B**) The design includes the self-induced Sal system combined with atsRNA to suppress *gfp* expression, resulting in the creation of StrainG-sRNA-*gfp*. (**C**) The characterization of StrainG-sRNA-*gfp* is presented, with the data representing mean ± SD from three independent experiments.

To validate the effectiveness of the system we built, we opted for utilizing the system to dynamically inhibit the growth-essential gene *ppc* and accumulate phosphoenolpyruvate (PEP), a precursor substance required for producing salicylic acid [1]. We selected the artificial trans-encoded sRNAs (atsRNAs) [33] as the repression mechanism. These atsRNAs consist of four components: the promoter, the target-binding sequence, the scaffold derived from *micC*, and the T1 transcriptional terminator (Figure 4A). The atsRNA consists of two parts: a scaffold sequence and a target binding sequence. atsRNAs in *E. coli* contain a consensus secondary structure that provides a scaffold for recruitment of Hfq proteins, which facilitates hybridization of the atsRNA to the target mRNA and mRNA degradation [34].

The inhibitory effect of combining atsRNAs with the self-induced Sal system was initially confirmed in *E. coli*. A control strain, known as StrainG, was utilized to continuously express the reporter gene *gfp*. Building upon StrainG, the combination of P_sal_ and atsRNAs was implemented to suppress the expression of GFP, resulting in the construction of StrainG-MicC1 (Figure 4B). Characterization results demonstrated that the fluorescence intensity of StrainG-MicC1 was significantly lower compared to that of StrainG. These findings indicate that the self-induced Sal system, in conjunction with atsRNAs, exhibits notably positive effects in *E. coli* (Figure 4C).

The study of bacterial genomes has been significantly constrained by current techniques like homologous recombination and transposon mutagenesis for deciphering prokaryotic gene function. Moreover, traditional antisense RNAs have limited efficacy in inhibiting gene activity [33,35,36]. In contrast, atsRNA-mediated gene silencing offers numerous advantages, providing a straightforward and swift approach to down-regulate the expression of specific genes [34,37,38].

### 2.4. Application of the Sal System for the Production of Salicylic Acid

Salicylic acid is produced in *E. coli* by utilizing branched-chain amino acids in the shikimate pathway as the building blocks. Research has revealed that a sufficient supply of PEP is crucial for the biosynthesis of compounds derived from the shikimate pathway [39]. PEP not only acts as a precursor for the shikimate pathway, but is also an essential metabolite in glycolysis and the TCA cycle [40]. Deletion of the phosphoenolpyruvate carboxylase gene (*ppc*) in *E. coli* inhibits some glycolytic enzymes, such as Pgi and Pfk, and the key glycolytic enzyme hexokinase is thought to be allosterically inhibited by its product G6P [41]. More NADPH is produced in the *ppc* mutant, which is also considered to be the reason for the downregulation of 6PGDH. Transcripts of the glucose transport gene *ptsG* are also associated with the accumulation of glycolytic intermediates such as G6P and F6P, which degrade *ptsG* mRNA by activating the RNaseP enzyme [42]. Downregulation of both glycolysis and pentose phosphate pathway enzymes results in slower growth rates and reduced glucose uptake rates in *ppc* mutants. Therefore, to produce salicylic acid, a self-induced Sal system combined with atsRNA is employed to dynamically repress the *ppc* gene. The atsRNA targeting *ppc* gene is expressed under the control of the P_sal_ promoter to form atsRNA with a secondary structure, which provides a scaffold for recruiting the Hfq protein facilitating the hybridization of atsRNA and mRNA (transcript product of the *ppc* gene) and the degradation of mRNA (transcript product of the *ppc* gene).

**Figure 5:**
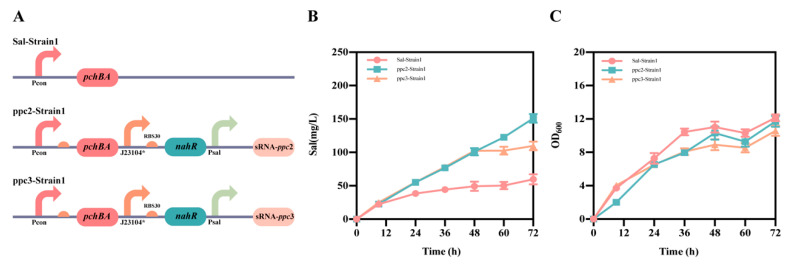
Application of the Sal system in the production of salicylic acid. (**A**) The design involved two strains named ppc2-Strain1 and ppc3-Strain1. (**B**) Salicylic acid titers of Sal-Strain1, ppc2-Strain1 and ppc3-Strain1 strains. (**C**) Growth of Sal-Strain1, ppc2-Strain1 and ppc3-Strain1 strains. The data shown represent the mean ± SD from three independent experiments.

The pSal1 plasmid, which serves as the foundation for salicylic acid production, was created by expressing the *pchBA* genes on a high-copy plasmid in a constitutive manner (as shown in Figure S1). For assessing the efficacy of different target-binding sequences in suppressing the *ppc* gene, we selected two such sequences known as ppc2 and ppc3 (as indicated in Table S5). We constructed plasmids pSal1-ppc2 and pSal1-ppc3 to regulate the *ppc* gene dynamically using the Sal system on plasmid Sal1. The *E. coli* TOP 10 strain was transformed with these plasmids, resulting in three strains: Sal-Strain1, ppc2-Strain1, and ppc3-Strain1 (as shown in Figure 5A). Subsequently, shake flask fermentation experiments were conducted on these strains, and the findings are presented in Figure 5B,C. Although the growth curves for the three strains were similar, there was a notable disparity in the production of salicylic acid. After 48 h of fermentation, Sal-strain1, ppc2-strain1, and ppc3-strain1 had salicylic acid titers of 47 mg/L, 100 mg/L, and 99 mg/L, respectively, with no significant difference in the titers between Sal-strain1 and ppc3-strain1. After 72 h of fermentation, the salicylic acid titers for Sal-Strain1, ppc2-Strain1, and ppc3-Strain1 were 60 mg/L, 151 mg/L, and 100 mg/L, respectively. The final results demonstrated a 151% increase in salicylic acid titer in ppc2-Strain1 compared to the Sal-Strain1 upon applying the Sal system to down-regulate the *ppc* gene dynamically, and a 66% increase in the salicylic acid titer in ppc3-Strain1 compared to Sal-Strain1. The discrepancy in salicylic acid production between ppc2-Strain1 and ppc3-Strain1 may be attributed to varying levels of repression in the *ppc* gene, with ppc3-Strain1 possibly accumulating more PEP to enhance salicylic acid production. These findings validate the effectiveness of the self-induced Sal system and highlight its potential in the field of metabolic engineering.

## 3. Conclusions

The development of a new orthologous QS system based on natural QS systems is crucial for synthetic biology research. This study successfully constructs a completely orthogonal self-induced Sal system in *E. coli*, adding to the repertoire of synthetic biology tools available. Moreover, the system was utilized in the production of salicylic acid, resulting in a 151% increase in titer compared to the control bacterium. These findings demonstrate the effectiveness of the system and its potential applications in metabolic engineering.

## 4. Materials and Methods

### 4.1. Culture Media and Salicylic Acid Fermentation

Luria–Bertani (LB) broth, containing a 5 g/L yeast extract, a 10 g/L tryptone, and a 10 g/L NaCl, was utilized for plasmid cloning, while LB agar (supplemented with a 15 g/L agar powder) was used for plasmid construction. The M9C medium consisted of a 20 g/L glycerol, a 2.5 g/L glucose, a 5 g/L yeast extract, a 6 g/L Na_2_HPO_4_, a 0.5 g/L NaCl, a 3 g/L KH_2_PO_4_, a 1 g/L NH_4_Cl, a 2 g/L MOPS, a 14.7 mg/L CaCl_2_·H_2_O, a 246.5 mg/L MgSO_4_·7H_2_O, a 0.25 mg/L CuSO_4_·5H_2_O, a 2 mg/L VB1, a 1.25 mg/L H_3_BO_3_, a 0.7 mg/L CoCl_2_·6H_2_O, a 1.6 mg/L MnCl_2_·4H_2_O, a 0.3 mg/L ZnSO_4_·7H_2_O, and a 0.15 mg/L NaMoO_4_·2H_2_O. Shake-flask fermentation involved culturing single colonies in fresh LB medium at 37 °C for approximately 12 h. The cultured seeds were then transferred to a 300 mL shake flask equipped with flaps, containing 50 mL of M9C medium with a 1% (*v*/*v*) inoculation. Fermentation was carried out at 220 rpm and 37 °C for 72 h. Antibiotics (chloramphenicol at 17 μg/mL and ampicillin at 100 μg/mL) were used to maintain plasmids and screen recombinants. Inducers like salicylic acid (W398500; Sigma-Aldrich, Shanghai, China) were added if necessary.

### 4.2. Strains and Plasmid Construction

*E. coli* DH5α was utilized for constructing plasmids, while *E. coli* TOP 10 was used for characterizing fluorescence, constructing cascade circuits, and biosynthesizing salicylic acid. Supplementary Table S1 lists the other strains used. Plasmids and primers mentioned in this article are listed in Supplementary Tables S2 and S3, respectively. Primers were synthesized for plasmid construction through PCR and Gibson assembly [43]. Plasmid cloning was performed in LB broth, while plasmid construction was carried out on LB agar. Antibiotics (chloramphenicol at 17 μg/mL and ampicillin at 100 μg/mL) were used to maintain plasmids and screen recombinants. Salicylic acid (W398500; Sigma-Aldrich, Shanghai, China) was added if inducers were required.

The main plasmids utilized in our study were pSalA and pGFP-100. The pSalA vector was derived from pTra*, which was originally created by Jiang et al. [20]. Primers B-pTra*-R and B-pTra*-F were used to obtain the linear backbone fragment using pTra* as a template, Psal-F and Psal-R to obtain the P_sal_ sequence, and P_sal_ was assembled upstream of *gfp* using Gibson assembly to obtain the pSalA plasmid. Primers B-pSalA-R and B-pSalA-F were used to obtain the linear backbone fragment using pSalA as a template. Promoter J23100 was included in the primers. Promoter J23100 was assembled upstream of *gfp* using the Gibson assembly method to obtain the pGFP-100 plasmid. The *pchBA* and *nahR* genes were cloned from *P. aeruginosa* and *Pseudomonas putida*, respectively, both of which were obtained from the culture collection in the State Key Laboratory of Microbial Technology at Shandong University. As for J23100, J23104 and J23104*, they were included in the upstream primers of genes *pchBA* and *nahR* for assembly. Additionally, the pSalC plasmid was based on the pSalA plasmid as a template, using primers B-pSalC-1 and B-pSalC-2 to obtain the fragment containing chloramphenicol and P_sal_-RBS30. Primers pchBA-F and pchBA-R were used to obtain the *pchBA* gene fragment, and primers B-pSalC-3 and B-pSalC-4 were to obtain the backbone fragment containing the ColE1 replicon. Primers nahR-F and nahR-R were used to obtain the *nahR* gene fragment, and the partial promoter J23100 fragment of the *pchBA* genes was included in primer B-pSalC-3. The partial promoter J23104 fragment of the *nahR* gene was included in primer B-pSalC-4, and the plasmid pSalC was obtained by Gibson assembly. In order to optimize the experiments, we replaced the wild-type RBS before *gfp* and *nahR* with four different RBS options, namely BBa-B0030, BBa-B0034, and BBa-B0035 (available at http://parts.igem.org/Main_Page, accessed on 9 April 2021). These different RBS designs were incorporated into the corresponding DNA fragments using primers and the DpnI digestion method to form pSalB-1, pSalB-2, pSalB-3, and pSalC. Furthermore, the ppc2 gene fragment was synthesized by gene synthesis (Tsingke, Qingdao, China). Primers B-ppc2-R and B-ppc2-F were used to obtain the linear backbone sequence using plasmid pSalC as a template, and primers ppc2-R and ppc2-F were used to obtain the ppc2 fragment. The synthetic sRNA sequence of the ppc2 site was assembled by Gibson to obtain the plasmid pSal1-ppc2. Primers B-ppc3-R and B-ppc2-F were used to obtain the linear backbone sequence using plasmid pSal1-ppc2 as a template. Primers ppc2-R and ppc3-F were used to obtain the synthetic sRNA sequence targeting the ppc3 site. After Gibson assembly, plasmid pSal1-ppc3 was obtained. The ppc2 and ppc3 fragments were assembled into the pSalC construct strains pSal1-ppc2 and pSal1-ppc3 using the Gibson method.

### 4.3. PCR: Amplifying Files

The reaction followed a thermal protocol: (1) 95 °C for 10 min, (2) 95 °C for 30 s, (3) 55 °C 2 k/min, (4) 72 °C for 30 s, (5) 72 °C for 10 min, (6) storage at 16 °C and performance of 30 cycles of steps (2)–(4). Confirmation of gene insertion and gene size with promoter on a 0.8% (*w*/*v*) agarose gel and confirmation of the size of the PCR product using a CLiN fragment analyzer. Employment of Phanta Max Master DNA polymerase for gene cloning and 2 × Taq Master Mix (Dye Plus) for colony PCR verification (Vazyme, Nanjing, China). Selection of positive clones on LB agar plates containing chloramphenicol and verification by PCR analysis.

### 4.4. Sal System Characterization

*E. coli* TOP 10 cells transformed with plasmid constructs were cultured for measuring in vivo fluorescence expression as follows: single colonies were transferred to 5 mL of LB broth with appropriate antibiotics. They were then cultured at 220 rpm and 37 °C for 12 h. Next, 2% of the resulting cultures were inoculated into a 24-well microassay plate containing 2 mL of the LB medium supplemented with the necessary antibiotics. Different concentrations of inducer salicylic acid were added to the medium. The plate was then cultured at 37 °C with vigorous shaking. After a 6-h inoculation, the cell density at OD_600_ and the green fluorescence (excitation at 485 nm and emission at 528 nm) were measured using a Multi-Detection Microplate Reader (Synergy HT, Biotek, Winooski, VT, USA) after diluting and washing the cells.

### 4.5. Analytical Methods

The salicylic acid standards were purchased from Sigma-Aldrich (Shanghai). The LC-20AT HPLC system from Shimadzu in Kyoto, Japan equipped with a UV–vis detector and a reverse-phase Diamonsil C18 column (Diamonsil 5 μm, 250 mm × 4.6 mm) was used to quantify the samples and standards for salicylic acid. Methanol (100%) was used as Solvent A, while Solvent B was water with 0.1% formic acid. The column temperature was set at 35 °C and the injection volume was 20 μL. The mobile phase flowed at a rate of 0.8 mL/min with a gradient concentration: a 95 to 30% Solvent B for 20 min, followed by a 30 to 95% Solvent B for 5 min, and finally maintaining a 95% Solvent B for an additional 5 min. The quantification of salicylic acid was performed based on the peak areas from UV absorbance at 303 nm.

### 4.6. Statistical Analysis

The results were presented as the mean ± SEM. A one-way ANOVA was used to evaluate the differences between the means. A statistical significance level of *p* < 0.05 was considered.

## Data Availability

Data are contained within the article and Supplementary Materials.

## Supplementary Materials

The following supporting information can be downloaded at: https://www.mdpi.com/article/10.3390/molecules28237825/s1. In this study, Supplementary Table S1: Strains used in this study; Table S2: Plasmids used in this study; Table S3: Primers used in this study; Table S4: The detailed promoter information; Table S5: Sequences used in this study and Figure S1: Plasmid map of plasmid pSal1.are provided. These tables include information on strains, plasmids, primers, the mutated 23104* promoter sequence, and the binding sequence targeting the *ppc* gene.
