# Supplementary material for: The Construction of the Self-Induced Sal System and Its Application in Salicylic Acid Production
Source: Molecules. 2023 Nov 28;28(23):7825. doi: 10.3390/molecules28237825 (PMC10708014; doi:10.3390/molecules28237825)
Supplement: Supplementary file 1 [file molecules-28-07825-s001.zip › molecules-2702026-supplementary.pdf]

Supplementary materials

Table S1. Strains used in this study.

**Strains**	**Description**	**Source**
E. coli TOP10	*F- mcrAΔ(mrr-hsdRMS-mcrBC) φ80lacZΔM15ΔlacX74nupG recA1 araD139Δ(ara-leu)7697 galE15 galK16 rpsL(StrR) endA1 λ-*	Lab stock
SalA	*E. coli* TOP10 harboring pSalA-30	This study
SalB-1	*E. coli* TOP10 harboring pSalB-1	This study
J23104-30-*gfp*	*E. coli* TOP10 harboring pJ23104-30-*gfp*	This study
J23104-35-*gfp*	*E. coli* TOP10 harboring pJ23104-35-*gfp*	This study
J23104*-30-*gfp*	*E. coli* TOP10 harboring pJ23104*-30-*gfp*	This study
SalB-2	*E. coli* TOP10 harboring pSalB-2	This study
SalB-3	*E. coli* TOP10 harboring pSalB-3	This study
SalC	*E. coli* TOP10 harboring pSal-C	This study
StrainG-sRNA-*gfp*	*E. coli* TOP10 harboring pGFP-MicC1	This study
Sal-Strain1	*E. coli* TOP10 harboring pSal1	This study
ppc2-Strain1	*E. coli* TOP10 harboring pSal1-ppc2	This study
ppc3-Strain1	*E. coli* TOP10 harboring pSal1-ppc3	This study

Table S2. Plasmids used in this study.

**Plasmids**	**Description**	**Source**
pSalA	CmR, P_{sal}-RBS 30-*gfp*, ori ColE1	This study
pGFP-100	CmR, BBa_J23100-RBS 30-*gfp*, ori ColE1	This study
pSalB-1	CmR, BBa_J23104-RBS 30-*nahR*, P_{sal}-RBS 30-*gfp*, ori ColE1	This study
pJ23104-30-*gfp*	CmR, BBa_J23104-RBS 30-*gfp*, ori ColE1	This study
pJ23104-35-*gfp*	CmR, BBa_J23104-RBS 35-*gfp*, ori ColE1	This study
pJ23104*-30-*gfp*	CmR, BBa_J23104-RBS 30-*gfp*, ori ColE1	This study
pSalB-2	CmR, BBa_J23104-RBS 35-*nahR*, P_{sal}-RBS 30-*gfp*, ori ColE1	This study
pSalB-3	CmR, BBa_J23104*-RBS 30-*nahR*, P_{sal}-RBS 30-*gfp*, ori ColE1	This study
pSalC	CmR, BBa_J23100-RBS 34-*pchBA*, BBa_J23104*-RBS 30-*nahR*, P_{sal}-RBS 30-*gfp*, ori ColE1	This study
pGFP-MicC1	CmR, BBa_J23100-RBS 34-*pchBA*, BBa_J23104*-RBS 30-*nahR*, Psal-MicC1-*gfp*, ori ColE1	This study
pSal1	CmR, BBa_J23100-RBS 34-*pchBA*, ori ColE1	This study
pSal1-ppc2	CmR, BBa_J23100-RBS 34-*pchBA*, BBa_J23104*-RBS 30-*nahR*, Psal-MicC1-ppc2, ori ColE1	This study

pSal1-ppc3	CmR, BBa_J23100-RBS 34-*pchBA*, BBa_J23104*-RBS 30-*nahR*, Psal-MicC1-ppc3, ori ColE1	This study

Table S3. Primers used in this study.

Primer name	Sequences (5'-3')
B-pTra*-R	GTGATGACGGTGAAAACCTCTGAC
B-pTra*-F	CTAGAGATTAAAGAGGAGAAATACTAGATGCGTAAAGGAGAAGAACT
Psal-F	GCATGTGTCAGAGGTTTTCACCGTCATCACGGGGCCTCGCTTGGGTTATT
Psal-R	CATCTAGTATTTCTCCTCTTTAATCTCTAGGATCCTCTATGGTACTCGTG
B-pSalA-R	CTAGgctagcactgtacctaggactgagctagccgtcaaGTGATGACGGTGAAAACCTC
B-pSalA-F	agctcagtcctaggtacagtgctagcCTAGAGATTAAAGAGGAGAAATACTAGATGCGTAAAGGAGAAGAACT
B-pSalC-1	GAAAAAATCCGAGCCGATCA
B-pSalC-2	aaacgaagatcaaaacgaccgagcgc
B-pSalC-3	TCTTTCTCTAGAgctagcactgtacctaggactgagctagccgtcaaGATGGTAAGCCCTCCCGTAT
B-pSalC-4	TAATCTCTAGAGCTAGCACAATACCTAGGACTAGCTAGCTGTCAAGTGATGACGGTGAAAACCTC
nahR-R	CCCATCACATTGATCGGCTCGGATTTTTTCTTAATCGGTAAACAGATCAAAC
pchBA-F	cgctgcgctcggtcgttttgatcttcgttttcaggcgacgccgcgctgca
pchBA-R	cctaggtacagtgctagcTCTAGAGAAAGAGGAGAAATACTAGATGgctatgaaaactcccgaagactg
nahR-F	TCCTAGGTATTGTGCTAGCTCTAGAGATTAAAGAGGAGAATACTAGATGATGGAACTGCGTGATCTGGA
B-ppc2-R	GATCCTCTATGGTACTCGTG
B-ppc2-F	ttaagaccgccggtcttgtc
ppc2-R	caaggtagtggacaagaccggcggtcttaaTATAAACGCAGAAAGGCCCA
ppc2-F	ATAAAGCCATCACGAGTACCATAGAGGATCatgaacgaacaatattccgc
B-ppc3-R	CAGAAAcctttcggcgaaatgctgtggtatGATCCTCTATGGTACTCGTG
ppc3-F	ataccacagcatttcgccgaaaggTTTCTGTTGGGCCATTGCAT

Table S4. The detailed promoter information.

Promoters	Sequences (5’-3’)
BBa_J23100	TTGACGGCTAGCTCAGTCCTAGGTACAGTGCTAGC
P_{sal}	GGGGCCTCGCTTGGGTTATTGCTGGTGCCCGGCCGGGCGCAA<br>TATTCATGTTGATGATTTATTATATATCGAGTGGTGTATTTATCA<br>ATATTGTTTGCTCCGTTATCGTTATTAACAAGTCATCAATAAAG<br>CCATCACGAGTACCATAGAGGATC
BBa_J23104	TTGACAGCTAGCTCAGTCCTAGGTATTGTGCTAGC
BBa_J23104*	TTGACAGCTAGCTAGTCCTAGGTATTGTGCTAGC

Table S5. Sequences used in this study.

Genes	Sequences (5’-3’)
pchBA	atgaaaactcccgaagactgcaccggcctggcggacatccgcgaggccatcgaccggatcgacct<br>ggatatcgtccaggccctcggccgccgcatggactacgtcaaggcggcgtcgcgcttcaaggccag<br>cgaggcggcgattccggcgcccgagcgggtcgccgcgatgctccccgagcgcgcccgctgggcc<br>gaggaaaacggactcgacgcgcccttcgtcgagggactgttcgcgcagatcatccactggtacatcg<br>ccgagcagatcaagtactggcgccagacacggggtgccgcatgagccggctggcgcccctgagcc<br>agtgcctgcacgccttgcgcggcaccttcgagcgcgccatcggccaggcgcaggcgctcgatcgtc<br>cggtgctggtggcggcatcgttcgagatcgacccattggacccgctgcaggtattcggtgcctggga<br>cgaccggcaaacgccctgcctgtactgggaacagcccgagctggcgttcttcgcctggggctgcgc<br>cctggagctgcaaggccacggcgaacagcgcttcgcccggatcgaggaaaactggcaattgctctg<br>cgccgacgccgtggtcgagggcccgctggcgccgcgcctgtgcggcggattccgcttcgatccgc<br>gcggcccgcgcgaggaacactggcaagccttcgccgatgccagcctgatgctcgccggcatcacc<br>gtgctgcgcgagggcgaacgctaccgggtactctgccaacacctggccaagcccggcgaagatgc<br>cctggccctggccgcctaccactgctcggcgctactgcgcctgaggcagccggccagacgccggc<br>cctcggggccgaccgctggcgcgcagggcgacgcttcggcgcaggagcgcaggcaatgggaag<br>ccaaggtgagcgacgcggtaagcagtgtccgccagggacgcttcggcaaggtcgtgctggcccgc<br>acccaggcccggcctctcggcgacatcgagccgtggcaggtcatcgaacacctgcgtctgcaacat<br>gccgacgcccagctgttcgcctgtcgccgcggcaacgcctgcttcctcggcgcctccccggaacgc<br>ctggtccgcattcgcgccggcgaggcactcacccatgccctggccgggaccatcgcccgcggcgg<br>cgatgcccaggaagatgcgcggctcggacaggccctgctggacagcgccaaggacaggcacgaa<br>caccagttggtggtggaggcgatccgtacggccctggaacccttcagcgaggtgctggaaatcccc<br>gatgcgcccggcctgaaacgactggcgcgagtccagcacctgaacacgccgatccgcgcccgcct<br>cgctgacgcaggcggcatcctgcggctgctacaagcgctgcatccgacccccgcggtgggcggct<br>acccacgcagcgcggcgctggactacatccgccagcacgaagggatggaccgcggctggtacgc<br>cgcgccgctgggctggctcgacggcgaaggcaacggcgatttcctggtggcgctgcgctcggccct<br>gctcacgccgggccggggctacctgttcgccggctgcggtctggtaggcgattcggaaccggccca<br>cgagtatcgcgaaacctgccttaagctcagtgccatgcgggaagctctatccgccataggcggcctg<br>gacgaagtgcccttgcagcgcggcgtcgcctga
nahR	ATGGAACTGCGTGATCTGGATCTGAATCTGCTGGTTGTGTTTA<br>ATCAGCTGCTGGTGGATCGTCGCGTTAGTGTTACCGCAGAAAA<br>TCTGGGTCTGACCCAGCCGGCAGTGAGTAATGCACTGAAACG<br>TCTGCGCACCAGTCTGCAAGATCCGCTGTTTGTGCGTACCCAT<br>CAGGGCATGGAACCGACCCCGTATGCAGCACATCTGGCCGAA

CCTGTTACCAGCGCAATGCATGCCCTGCGCAATGCACTGCAAC
ATCATGAAAGTTTTGATCCGCTGACCAGTGAACGTACCTTTAC
CCTGGCAATGACCGATATTGGCGAAATCTATTTTATGCCGCGCC
TGATGGATGTTCTGGCCCATCAGGCACCGAATTGCGTTATTAG
CACCGTGCGTGATAGTAGCATGAGTCTGATGCAGGCCCTGCAA
AATGGCACCGTGGATCTGGCCGTTGGTCTGCTGCCGAATCTGC
AAACCGGCTTTTTCCAGCGTCGCCTGCTGCGTAATCATTATGTT
TGTCTGTGCCGTAAAGATCATCCGGTTACCCGTGAACCGCTGA
CCCTGGAACGCTTTTGTAGTTATGGTCATGTGCGTGTTATTGCA
GCCGGCACCGGCCACGGTGAAGTGGATACCTATATGACCCGC
GTTGGCATTCGTCGCGATATTCGTCTGGAAGTTCCGCATTTTGC
AGCAGTTGGTCATATTCTGCAACGCACCGATCTGCTGGCAACC
GTGCCGATTCGTCTGGCCGATTGCTGTGTGGAACCGTTTGGCC
TGAGTGCACTGCCGCATCCGGTGGTTCTGCCGGAAATTGCAAT
TAATATGTTTTGGCATGCAAAGTATCACAAAGATTTGGCAAATA
TTTGGCTGCGTCAGCTGATGTTTGATCTGTTTACCGATTAA

gfp	ATGCGTAAAGGAGAAGAACTTTTCACTGGAGTTGTCCCAATTC<br>TTGTTGAATTAGATGGTGATGTTAATGGGCACAAATTTTCTGTC<br>AGTGGAGAGGGTGAAGGTGATGCAACATACGGAAAACTTACC<br>CTTAAATTTATTTGCACTACTGGAAAACTACCTGTTCCATGGCC<br>AACACTTGTCACTACTTTCGGTTATGGTGTTCAATGCTTTGCGA<br>GATACCCAGATCATATGAAACAGCATGACTTTTTCAAGAGTGC<br>CATGCCCGAAGGTTATGTACAGGAAAGAACTATATTTTTCAAA<br>GATGACGGGAACTACAAGACACGTGCTGAAGTCAAGTTTGAA<br>GGTGATACCCTTGTTAATAGAATCGAGTTAAAAGGTATTGATTT<br>TAAAGAAGATGGAAACATTCTTGGACACAAATTGGAATACAA<br>CTATAACTCACACAATGTATACATCATGGCAGACAAACAAAAG<br>AATGGAATCAAAGTTAACTTCAAAATTAGACACAACATTGAAG<br>ATGGAAGCGTTCAACTAGCAGACCATTATCAACAAAATACTCC<br>AATTGGCGATGGCCCTGTCCTTTTACCAGACAACCATTACCTG<br>TCCACACAATCTGCCCTTTCGAAAGATCCCAACGAAAAGAGA<br>GACCACATGGTCCTTCTTGAGTTTGTAACAGCTGCTGGGATTA<br>CACATGGCATGGATGAACTATACAAATAA
ppc2	GGGGCCTCGCTTGGGTTATTGCTGGTGCCCGGCCGGGCGCAAT<br>ATTCATGTTGATGATTTATTATATATCGAGTGGTGTATTTATCAAT<br>ATTGTTTGCTCCGTTATCGTTATTAACAAGTCATCAATAAAGCC<br>ATCACGAGTACCATAGAGGATCATGAACGAACAATATTCCGCA<br>TTGTTTCTGTTGGGCCATTGCATTGCCACTGATTTTCCAACATA<br>TAAAAAGACAAGCCCGAACAGTCGTCCGGGCTTTTTTctcgagC<br>TCGAGCCAGGCATCAAATAAAACGAAAGGCTCAGTCGAAAGA<br>CTGGGCCTTTCGTTTTATCTGTTTTTGTCGGTGAACGCTCTCTA<br>CTAGAGTCACACTGGCTCACCTTCGGGTGGGCCTTTCTGCGTT<br>TATA

ppc3	GGGGCCTCGCTTGGGTTATTGCTGGTGCCCGGCCGGGCGCAAT<br>ATTCATGTTGATGATTTATTATATATCGAGTGGTGTATTTATCAAT<br>ATTGTTTGCTCCGTTATCGTTATTAACAAGTCATCAATAAAGCC<br>ATCACGAGTACCATAGAGGATCATACCACAGCATTTCGCCGAA<br>AGGTTTCTGTTGGGCCATTGCATTGCCACTGATTTTCCAACATA<br>TAAAAAGACAAGCCCGAACAGTCGTCCGGGCTTTTTTTctcgagC<br>TCGAGCCAGGCATCAAATAAAACGAAAGGCTCAGTCGAAAGA<br>CTGGGCCTTTCGTTTTATCTGTTTTTGTCGGTGAACGCTCTCTA<br>CTAGAGTCACACTGGCTCACCTTCGGGTGGGCCTTTCTGCGTT<br>TATA

The atsRNA is expressed under the control of the P_{sal} promoter (yellow sequence). The atsRNA target-binding sequence is inserted into the red region, which is 24 nucleotides long. Reliable inhibition of atsRNA is achieved by additional sequences (green sequences) bound by Hfq. The remaining part is the transcription terminator (T1/TE).

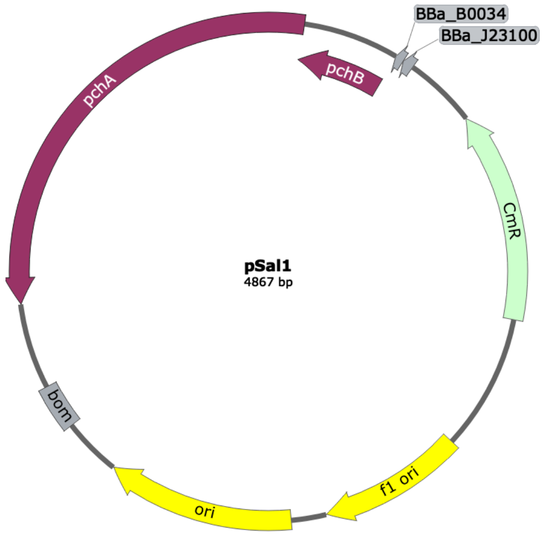

Figure S1. Plasmid map of plasmid pSal1. The vector of plasmid pSal1 is pETDuet-1, ColE1 replicon, chloramphenicol resistance, and the *pchBA* genes are constitutively expressed under the control of the BBa_J23100 promoter sequence and the BBa_B0034 RBS sequence.
